# The Autophagic and Apoptotic Death of Forebrain Neurons of Rats with Global Brain Ischemia Is Diminished by the Intranasal Administration of Insulin: Possible Mechanism of Its Action

**DOI:** 10.3390/cimb46070392

**Published:** 2024-06-27

**Authors:** Irina O. Zakharova, Liubov V. Bayunova, Daria K. Avrova, Alina D. Tretyakova, Alexander O. Shpakov, Natalia F. Avrova

**Affiliations:** Sechenov Institute of Evolutionary Physiology and Biochemistry, Russian Academy of Sciences, Thorez Av. 44, St. Petersburg 194223, Russia; zakhar@iephb.ru (I.O.Z.); bayunoval@mail.ru (L.V.B.); avrovacat@mail.ru (D.K.A.); alina_tretyakova_2004@list.ru (A.D.T.); alex_shpakov@list.ru (A.O.S.)

**Keywords:** forebrain ischemia, intranasal insulin, autophagy, apoptosis, neuronal viability, protein kinases

## Abstract

Insulin is a promising neuroprotector. To better understand the mechanism of insulin action, it was important to show its ability to diminish autophagic neuronal death in animals with brain ischemic and reperfusion injury. In forebrain ischemia and reperfusion, the number of live neurons in the hippocampal CA1 region and frontal cortex of rats decreased to a large extent. Intracerebroventricular administration of the autophagy and apoptosis inhibitors to ischemic rats significantly increased the number of live neurons and showed that the main part of neurons died from autophagy and apoptosis. Intranasal administration of 0.5 IU of insulin per rat (before ischemia and daily during reperfusion) increased the number of live neurons in the hippocampal CA1 region and frontal brain cortex. In addition, insulin significantly diminished the level of autophagic marker LC3B-II in these forebrain regions, which markedly increased during ischemia and reperfusion. Our studies demonstrated for the first time the ability of insulin to decrease autophagic neuronal death, caused by brain ischemia and reperfusion. Insulin administered intranasally activated the Akt-kinase (activating the mTORC1 complex, which inhibits autophagy) and inhibited the AMP-activated protein kinase (which activates autophagy) in the hippocampus and frontal cortex of rats with brain ischemia and reperfusion.

## 1. Introduction

The increase in human longevity has been associated with the improvement of the treatment of numerous diseases. Cerebrovascular pathologies are frequently mentioned as the cause of death of millions of people, which is confirmed by the World Health Organization [1]. According to “The Global Burden of Diseases Study”, which included more than 200 countries and comprised the period from 1990 to 2019 years, brain ischemia ranks third place in the world among the reasons for premature death and disability. The results of a systemic study showed that the amount of cerebral stroke deaths in the world has risen, with more people dying in developing countries than in developed ones [2]. Moreover, in developed countries, ischemic brain lesions often lead to long-term and severe disability [3]. One of the pathological conditions that significantly enhances the possibility of cerebral strokes, including ischemic ones, is cerebral small vessel disease (CSVD), which leads to brain tissue damage and impairment of cognitive functions [4,5]. At present, major attention is paid to the investigation of its manifestations and pathogenesis, to various approaches to the therapy and protection against this pathology [4,5,6]. Identification of neuroprotectors that can have a beneficial effect during ischemia and reperfusion injury of the brain appears to be an urgent task, as it may result in the subsequent introduction of these compounds into clinical practice.

Insulin possesses both hormonal and neurotrophic factor activities. It belongs to the group of neuroprotectors that are promising for clinical use. Insulin is a peptide compound that is able to directly penetrate the brain, bypassing the blood–brain barrier in the case of its intranasal [7,8] or intracerebroventricular (ICV) administration. The non-traumatic intranasal way of insulin delivery was successfully applied in clinical trials to treat patients with Alzheimer’s disease, Parkinson’s disease and other neurodegenerative disorders [9,10,11]. The neuroprotective effects of insulin have previously been studied in animal models of various neurodegenerative pathologies [12,13].

However, there are only a few works devoted to the studies of the protective effect of insulin administered intranasally to animals with cerebrovascular disorders [14,15,16]. The results disclosing the ability of insulin to prevent autophagic death of brain neurons during brain ischemic and reperfusion injury or other cerebrovascular disorders are practically absent.

Currently, the ideas about the exclusively protective action of autophagy, which plays a key role in maintaining cellular homeostasis, have been revised. The participation of autophagy in nerve cell death, especially in the death of neurons in the CA1 region of the hippocampus, which is known to be the most sensitive to injury [17], is clearly visible, in particular, during brain ischemia and reperfusion. The hippocampus (earlier named cornu Ammonis) consists of various subfields or regions, and CA1 is the first region in the hippocampal circuit. The ability of many neuroprotectors to increase the viability of brain neurons due to the decrease in their autophagic death during brain ischemia and reperfusion was demonstrated. These neuroprotectors include flavonoids, resveratrol, GM1 ganglioside, N-acetyl-serotonin (melatonin precursor), Fingolimod (sphingosine-1-phosphate analog) and other compounds [18,19,20,21,22,23,24].

At the same time, it should be noted that in certain cases the activation of autophagy in the ischemic and reperfused brain can be protective [25,26,27]. However, it seems that with sufficient strength of the ischemic brain injury in its acute phase, activation of autophagy leads to neuronal death. For this reason, it appears to be important to study the ability of promising neuroprotectors to prevent autophagic death of neurons in the pathological conditions associated with brain ischemia and reperfusion. Insulin appears to belong to the neuroprotectors, which have a chance to be used in the future in clinical practice for the treatment of such pathologies.

The goal of this work was to estimate the autophagic and apoptotic death of nerve cells in the hippocampal CA1 region and frontal cerebral cortex of rats exposed to global forebrain ischemia and subsequent reperfusion and the ability of intranasally administered insulin to diminish it. The ability of insulin and inhibitors of autophagy—3-methyladenine (3-MA) and apoptosis (acetyl-aspartyl-glutamyl-valyl-aspartal—Ac-DEVD-CHO)—to change the levels of autophagic marker LC3B-II and glial fibrillary acidic protein (GFAP) increased in the hippocampus and frontal cerebral cortex during ischemia, and reperfusion was also studied. In order to try to elucidate the possible mechanism of insulin action, its effect on the activities of protein kinases, which determine the intensity of autophagy and apoptosis, was investigated in the forebrain structures.

## 2. Materials and Methods

### 2.1. The Experimental Design and Animals Used

Experiments were carried out on adult male Wistar rats weighing 270–330 g. Two-vessel forebrain ischemia was induced in rats by clamping the carotid arteries for 10 min, in combination with lowering blood pressure to 50 mm.

In the experiments devoted to the determination of the number of live neurons in the CA1 region of the hippocampus and frontal cortex reperfusion was performed for 7 days. Bovine insulin (#I5500, Sigma-Aldrich, St. Louis, MO, USA) at a dose of 0.5 IU per rat was administered intranasally 1 h before occlusion of the carotid arteries and hypotension, and then daily during the reperfusion stage. In order to determine how the activation of autophagy and apoptosis affects the viability of neurons during two-vessel forebrain occlusion with hypotension and subsequent reperfusion, autophagy (3-methyladenine—3-MA, #M9281, Sigma-Aldrich, St. Louis, MO, USA) and apoptosis (Ac-DEVD-CHO, #556465, BD Pharmigen, BD Biosciences, San Diego, CA, USA) inhibitors were administered ICV in the brain of rats 0.5 h before the onset of ischemia. Sham-operated rats were used as control animals. In this series of experiments, 8 groups of animals were studied: sham-operated rats (Sh-O), Sh-O + insulin, Sh-O + 3-MA, Sh-O + Ac-DEVD-CHO, rats with brain ischemia and reperfusion (I-R), I-R + insulin, I-R + 3-MA and I-R + Ac-DEVD-CHO.

Several series of experiments were devoted to the determination of the effect of ischemia and reperfusion, of the administration of insulin (intranasally) and inhibitors of autophagy and apoptosis (ICV) on the level of the markers of autophagy and apoptosis (LC3B-II and the activity of caspase-3, respectively), of glial fibrillary acidic protein (GFAP) and on the intensity of insulin receptor substrate-2 (IRS-2) phosphorylation at Ser^731^ in the hippocampus and frontal cortex of rats. In all of these experiments, reperfusion was performed for 3 days. Insulin and inhibitors of autophagy and apoptosis were administered as indicated above, with daily administration of insulin being performed for 3 days. In all the above-mentioned series of experiments, 8 groups of animals were studied: Sh-O, Sh-O + insulin, Sh-O + 3-MA, Sh-O + Ac-DEVD-CHO, I-R, I-R + insulin, I-R + 3-MA and I-R + Ac-DEVD-CHO. In order to evaluate the activity of Akt-kinase, the level of pAkt (Ser^473^)/Akt was measured in the hippocampus and frontal cortex of rats after an ischemic episode and 2 h of reperfusion. In these experiments, 4 groups of animals were studied: Sh-O, Sh-O + insulin, I-R and I-R + insulin. In order to evaluate the activity of protein kinase AMPK, the level of pAMPK-alpha (Thr^172^)/AMPK-alpha was measured in the hippocampus and frontal cortex of rats after an ischemic episode and 3 days of reperfusion. Eight groups of animals were studied: Sh-O, Sh-O + insulin, Sh-O + 3-MA, Sh-O + Ac-DEVD-CHO, I-R, I-R + insulin, I-R + 3-MA and I-R + Ac-DEVD-CHO.

### 2.2. Surgery to Introduce Apoptosis and Autophagy Inhibitors into the Lateral (I) Ventricle of the Rat

In order to determine how the activation of autophagy and apoptosis affects the viability of neurons during two-vessel forebrain occlusion with hypotension and subsequent reperfusion, autophagy (3-MA) and apoptosis (Ac-DEVD-CHO) inhibitors were administered ICV in the brain of rats. In order to equalize the possible consequences of such administration, all rats in other groups of animals were injected with ICV with sterile phosphate buffer, which was used to dissolve the inhibitors. After anesthetizing the rats with chloral hydrate (#15307, Sigma-Aldrich, St. Louis, MO, USA), administered intramuscularly at a dose of 400 mg/kg, the animals were placed in stereotaxis. For craniotomy, a dental bur was used in the area located above the first ventricle of the brain (coordinates: AP = −0.92 mm, L = 1.5 mm, V = 3.5 mm relative to bregma) in accordance with the stereotactic atlas [28]. Rats were injected ICV with 20 μg of 3-MA (#M9281, Sigma-Aldrich, St. Louis, MO, USA) per rat, or 10 μg of Ac-DEVD-CHO, a specific caspase-3 inhibitor (#556465, BD Pharmigen, BD Biosciences, San Diego, CA, USA) per rat, dissolved in 4.5 μL of phosphate buffer, or a similar volume of phosphate buffer 30 min before ischemic exposure using a microsyringe (“Hamilton”, Reno, NV, USA).

### 2.3. Intranasal Administration of Insulin

Bovine insulin (#I5500, Sigma-Aldrich, St. Louis, MO, USA) at a dose of 0.5 IU per rat was administered intranasally 1 h before occlusion of the carotid arteries and hypo-tension, and then daily for 3 or 7 days at the reperfusion stage. To perform this test, 10 μL of a solution containing 1 mg of insulin in 1 mL of citrate buffer was collected into a 20 μL Sartorius pipette and instilled into one nostril of the rat, then another 10 μL of the solution was taken into the pipette and instilled into the other nostril of the same rat. Thus, the rat received a dose of insulin equal to 0.5 IU. The citrate buffer (pH 4.4) was prepared as a mixture of solutions of 100 mM citric acid (9.9 mL) and 100 mM sodium citrate (10.1 mL). We have chosen a dose of 0.5 IU of insulin per rat, as the preliminary experiments showed that the intranasal administration of insulin in such doses normalized important metabolic parameters such as the activity of Na+, K+-ATPase in the rat brain cortex, which was markedly and significantly decreased in the ischemic and reperfused brain. Intranasal administration of insulin in a dose of 0.5 IU increased Na+, K+-ATPase activity to the level found in the sham-operated rats, while the intranasal administration of insulin in a dose of 0.25 IU per rat did not result in such an effect and caused only small increase in the enzymatic activity. These results suggest the dose-dependent effects of insulin in forebrain ischemia and reperfusion. The data obtained in the present study of neuroprotective and metabolic effects of insulin administration to rats with forebrain ischemia and reperfusion provide further evidence that the dose of insulin equal to 0.5 IU is quite effective in the case of its intranasal administration. Much higher doses of insulin are usually used in the case of its systemic administration.

### 2.4. Two-Vessel Forebrain Ischemia with Hypotension and Subsequent Reperfusion

Two-vessel forebrain ischemia was induced in rats by clamping the carotid arteries for 10 min in combination with lowering blood pressure to 50 mm Hg by drawing blood into a syringe with heparin, as described previously [29]. The femoral artery was catheterized with a catheter (1.0 × 0.6 × 210 mm, 23G) purchased from “SciCat” company (Balashiha, Moscow Region, Russia). Forebrain reperfusion was carried out by decompressing the carotid arteries and returning blood with heparin, taken with a syringe at the stage of ischemia, into the bloodstream. Sham-operated rats were used as control animals. After completion of ischemia, the rats had their wounds treated with streptocide powder. The surgical incisions were sutured with sterile non-absorbable lavsan threads (MP1.5 UP4-0, Lintex LLC, Saint-Petersburg, Russia). Postoperative care of the rats was carried out for 3–7 days while keeping them on a standard diet.

### 2.5. Counting the Number of Live Neurons in the CA1 Region of the Hippocampus and Frontal Brain Cortex by Modified Nissl Method

Animals anesthetized with chloral hydrate were decapitated 7 days after the start of reperfusion. The brains were excised, washed with a chilled physiological solution and placed for 7 days in 4% paraformaldehyde in 10 mM phosphate buffer (+4 °C). For cryoprotection, the brain was kept in a solution of 30% sucrose in 10 mM PBS for 7 days at +4 °C, after which it was frozen in isopentane and then stored at −80°. Alternating series of frontal sections (10 μm) were obtained from the frontal cortex and CA1 region of the hippocampus using a cryostat (Leica Biosystems, Nussloch, Germany). Every 10th section was mounted on gelatin-coated glass (BioVitrum, Saint-Petersburg, Russia). To assess the number of live neurons, toluidine blue staining was performed using the modified Nissl method [30]. Five experiments were made in each group, and one rat was used in each experiment. The total number of measurements of live neurons in each experiment in various groups of animals was not less than 57. Images were taken using a Carl Zeiss Axio A1 microscope (Carl Zeiss, Jena, Germany) with a built-in AxioCam 712 color television camera (Carl Zeiss, Jena, Germany) and the Zen 3.4 Program (Zen pro). The number of viable cells in the hippocampal CA1 was counted over the region length of 300 μm, while in the cortex, across the square with a side of 300 μm, using Bio7 software version 2.5.0 (USA).

### 2.6. Caspase-3 Activity

The activity of caspase-3 in brain lysates was measured by a colorimetric Caspase 3 Assay kit (#ab39401, Abcam, Cambridge, UK) according to the manufacturer’s manual.

### 2.7. Evaluation by Immunoblotting of the LC3B-II and GFAP Level, Total Akt and AMPK-Alpha and Their Activities and the Inhibitory Phosphorylation of IRS-2 at Ser^731^ in Hippocampus and Frontal Cortex

Rats anesthetized with chloral hydrate were decapitated in 3 days after the start of reperfusion. The hippocampus and frontal brain cortex were dissected and homogenized in the ratio 1:20 in the lysis buffer containing 20 mM Tris-HCl (pH 7.5), 150 mM NaCl, 2 mM EGTA, 2 mM EDTA, 0.25% sodium deoxycholate, 0.5% Triton X-100, 15 mM NaF, 10 mM sodium glycerophosphate, 10 mM sodium pyrophosphate, 1 mM Na3VO4, 1 mM phenylmethylsulfonyl fluoride (PMSF), 0.02% NaN3 and the protease inhibitor cocktail (ServiceBio Technology, Wuhan, China). The cell fragments and the undamaged cells were separated by centrifugation at 500× *g* for 10 min (+4 °C). The protein concentration was measured by the Lowry method with BSA as a standard as previously described [31]. An amount of 20 µg of protein per sample was run on 9% or 13% SDS–polyacrylamide gel. The electro-phoresis of the samples was made at a constant voltage. Afterward, the proteins were transferred to 0.22 µm PVDF membrane (Bio-Rad, Hercules, CA, USA). The nonspecific binding sites of the membranes were blocked as it was previously described [31]. The level of autophagy marker protein LC3B-II was determined using specific antibodies to LC3B-II (1:1000, #2775, Cell Signaling Technology, Danvers, MA, USA). The level of GFAP showing the activation of astroglia was measured using mouse IgG2a antibodies raised against the KTVEMRDGEVIK sequence of GFAP (it was kindly gifted by the Federal Center of Brain and Neurotechnologies, Moscow, Russia). Monoclonal antibodies specific for pAkt (Ser^473^) (1:1000, #4058, Cell Signaling Technology, Danvers, MA, USA), for pAMPK alpha (Thr^172^) (1:1000, Cell Signaling Technology, Danvers, MA, USA) and pIRS-2 (Ser731) (#ab3690, Abcam, Cambridge, UK) were used to estimate the level of phosphorylation. The specific antibodies to total Akt (1:1000, #4691, Cell Signaling Technology, Danvers, MA, USA), total AMPK alpha (1:1000, #2793, Cell Signaling Technology, Danvers, MA, USA) and total IRS-2 (1:1000, #3089, “Cell Signaling Technology”, Danvers, MA, USA) were used to determine the level of these proteins and its possible changes. The membranes were incubated with primary antibodies at +4 °C overnight. Then, they were washed three times with 0.1% Tween 20 in Tris-buffered saline (50 mM Tris, pH 7.5, 150 mM NaCl) and treated with either anti-mouse (#7076) or anti-rabbit (#7074) HRP-IgG secondary antibody (Cell Signaling Technology, Danvers, MA, USA) diluted in 5% nonfat milk with TBST buffer for 1 h at room temperature. To normalize the data, the membranes were treated after stripping with the antibodies raised against glyceraldehyde 3-phosphate dehydrogenase (GAPDH) (1:3000, #T0004, Affinity Biosciences, Changzhou, China). Blots were developed with Novex ECL HRP enhanced chemiluminescence detection reagent Kit (Invitrogen, Waltham, MA, USA). The films were scanned on a CanoScan 8800F scanner (Canon, Tokyo, Japan). The optical densities of the positive bands on photo films were quantitated using Bio7 software (USA).

### 2.8. Statistical Analysis

Data are given as the mean ± SEM. The normality of distribution was tested by the Shapiro–Wilk test (Prizm 8). The statistical significance of the differences between the two groups of data was estimated by Student’s unpaired *t* test. The differences were considered significant at *p* < 0.05.

## 3. Results

**Figure 1:**
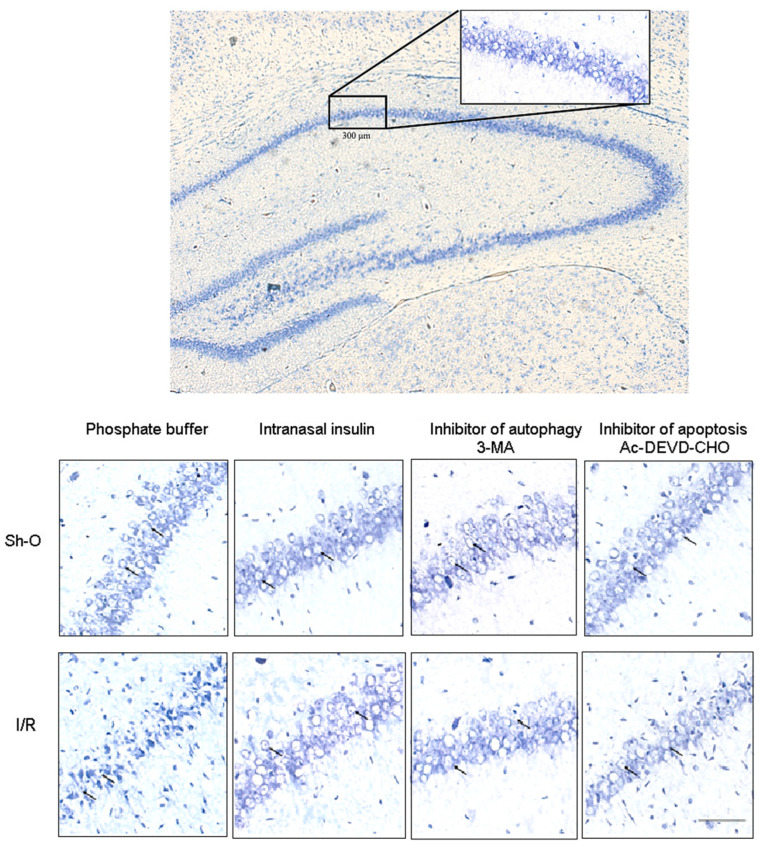
The effect of forebrain ischemia and subsequent reperfusion, insulin (intranasally administered) and autophagy or apoptosis inhibitors (3-MA and Ac-DEVD-CHO, respectively, administered ICV) on the number of live neurons in hippocampal CA1 region. Photographs of sections of hippocampal CA1 region were taken after staining by modified Nissl procedure in order to reveal the number of live neurons. Examples of live neurons with regularly shaped round cell bodies were marked with arrows. Scale 100 µm. Abbreviations used: Sh-O—sham-operated rats, I/R—rats exposed to brain ischemia and reperfusion, 3-MA—3-methyladenine.

Using a modified Nissl staining with toluidine blue, the number of live neurons in the hippocampal CA1 region was shown to be greatly diminished (almost two fold) when rats were exposed to forebrain ischemia and subsequent reperfusion for 7 days (Figure 1 and Table 1). Most parts of the nerve cells in the hippocampal CA1 region of sham-operated rats had regularly shaped round cell bodies, and such an appearance is characteristic of live neurons. In many of them, the nucleus and nucleolus inside could be seen. After ischemia and reperfusion, a large part of neurons in this brain region became shriveled, and they had irregularly shaped cell bodies and darker staining compared with that in the control group. The number of live neurons diminished from 46.8 ± 1.42 to 27.01 ± 3.23 live neurons/300 µm of the CA1 region of the hippocampus (*p* < 0.001, see Table 1). The results are given as the mean ± SEM from data obtained by measuring the number of live neurons in 5 separate experiments.

When insulin was administered intranasally to rats with an ischemic and reperfused brain, the number of live nerve cells in the CA1 region of the hippocampus increased to a great extent—from 27.01 ± 3.23 to 46.33 ± 2.90 live neurons/300 µm of CA1 region of the hippocampus (*p* < 0.01, Table 1). It almost reached the level of live neurons in the same brain region of sham-operated rats (46.80 ± 1.42 live neurons/300 µm). Thus, intranasal administration of insulin (before the onset of ischemia and then daily during 7 days of reperfusion) almost completely prevented the death of nerve cells in the hippocampal CA1 region in rats with brain ischemia and reperfusion.

ICV administration of the autophagy and the apoptosis inhibitors (3-MA and Ac-DEVD-CHO, respectively) was used. We could not reveal the significant effect of these inhibitors on the number of live neurons in the hippocampal CA1 region of sham-operated rats. In the hippocampal CA1 region of rats with ischemic and reperfused brains, the number of live neurons increased after the administration of these inhibitors. Thus, ICV administration of the autophagy inhibitor 3-MA to rats increased the number of live neurons in this brain region from 27.01 ± 3.23 to 38.81 ± 1.53 live neurons/300 µm of the CA1 region of the hippocampus (*p* < 0.02, see Table 1). The difference between these values is approximately equal to the number of nerve cells that died due to autophagy activation in the hippocampal CA1 region of rats with global ischemia and reperfusion. These data provide evidence that the activation of autophagy leads to the death of a significant proportion of all neurons that died from ischemia and reperfusion in the experimental conditions used.

The apoptotic process also makes a significant and pronounced contribution to the overall death of neurons during ischemia and subsequent reperfusion. Thus, ICV administration of Ac-DEVD-CHO, the apoptosis inhibitor, to rats increased the number of nerve cells surviving ischemia and subsequent reperfusion in the hippocampal CA1 region from 27.01 ± 3.23 to 35.33 ± 1.47 live neurons/300 µm (*p* < 0.05, Table 1). The difference between these values provides evidence about the approximate number of neurons that died from apoptosis. A comparison of the number of nerve cells that remained alive in the presence of autophagy and apoptosis inhibitors shows that the difference between these two values is not significant.

**Figure 2:**
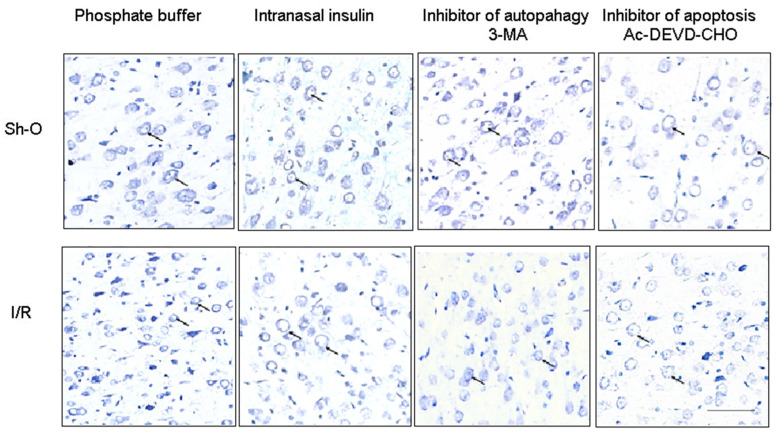
The effect of forebrain ischemia and subsequent reperfusion of insulin (administered intranasally) and autophagy or apoptosis inhibitors (3-MA and Ac-DEVD-CHO, respectively, administered ICV) on the number of live neurons in frontal brain cortex. Photographs of sections of frontal cortex were taken after staining by modified Nissl procedure in order to reveal the number of live neurons. Examples of live neurons with regularly shaped round cell bodies were marked with arrows. Scale 100 µm. Abbreviations used are given in Figure 1.

The neurons of the hippocampal CA1 region were shown to be more sensitive to ischemia and subsequent reperfusion compared to neurons of the frontal cortex (Figure 1 and Figure 2, Table 1). Thus, in the rat hippocampal CA1 region exposed to ischemia and reperfusion, 57.7% of neurons remained alive from their number in sham-operated rats, while in the rat frontal cortex, their number reached 72.5% of live neurons in the control brain (see Table 1).

The live neurons with regularly shaped round cell bodies were characteristic of the frontal brain cortex of sham-operated rats. In many of them, the nucleus and nucleolar within it could be seen. They markedly decreased in their number in this brain region of rats with global ischemia and reperfusion, from 67.29 ± 1.81 to 48.76 ± 2.33/standard area (300 µm × 300 µm) (*p* < 0.001, see Figure 2 and Table 1). Intranasal administration of insulin to rats enlarged the number of live neurons in the frontal cortex that survived ischemia and reperfusion from 48.76 ± 2.33 to 59.38 ± 1.16/standard area; the difference is quite significant (*p* < 0.01). At the same time, the latter value is significantly lower than the number of live neurons in the frontal cortex of control animals (*p* < 0.01, see Table 1). This means that in the frontal brain cortex, the neuroprotective effect of insulin against the ischemic and reperfusion injury was less pronounced than in the CA1 hippocampal region.

**Figure 3:**
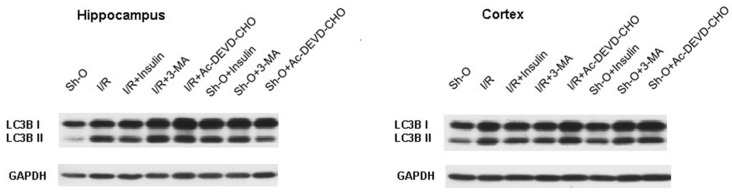
The effect of forebrain ischemia and subsequent reperfusion, insulin (administered intranasally), 3-MA and Ac-DEVD-CHO (administered ICV) on the LC3B-II level in rat hippocampus and frontal cerebral cortex (immunoblots). The quantitative data are summarized in Table 2. Abbreviations used are given in Figure 1.

It was also necessary to show that insulin is able to decrease the intensity of the autophagy processes in the forebrain regions of rats exposed to global ischemia and reperfusion. We studied the effect of ischemia and reperfusion and intranasally administered insulin on the level of LC3B-II. This substance is the lipidated form of LC3B (as opposed to the soluble form—LC3B-I). It is one of the main autophagic markers. We measured at different periods of reperfusion the levels of LC3B-II in hippocampal and cortical lysates of rats that received or did not receive insulin. We found that 3 days was the best period of reperfusion to reveal the effect of ischemia and reperfusion and insulin administration on the LC3B-II level. The main experiments were performed using this period of reperfusion after ischemic exposure. As one can see from the data shown in Figure 3 and Table 2, ischemia followed by reperfusion leads to an increase in the level of this autophagic marker, as shown by the calculation of both the LC3B-II to LC3B-I ratio and the LC3B-II to GAPDH ratio. These ratios were significantly increased by ischemia and reperfusion and decreased by ICV administration of the autophagy inhibitor 3-MA (20 μg per rat before brain ischemia) or by intranasal administration of insulin in a dose of 0.5 IU per rat (before the onset of ischemia and then every day during the reperfusion period, which lasted three days). The administration of insulin and 3-MA diminished the LCB3-II content in the hippocampus and frontal cerebral cortex of ischemic rats approximately to its content in the same brain region of sham-operated animals (Figure 3 and Table 2). These results clearly show the ability of insulin to diminish and normalize the level of this autophagy marker and, hence, the intensity of autophagic processes increased in the hippocampus and the cerebral cortex as a result of brain ischemia and reperfusion.

It was also necessary to see the effect of insulin on the caspase activity in the ischemic and reperfused brains and in the brains of sham-operated rats. In both forebrain structures studied, global ischemia and reperfusion significantly increased caspase-3 activity (Table 3). In the hippocampus, caspase activity diminished to the control level (*p* < 0.02) if insulin intranasal administration took place in rats with ischemic and reperfused brains. In the ischemic and reperfused brain cortex, the decrease in caspase-3 activity by intranasally administered insulin was also pronounced and significant. In sham-operated rats, insulin administration had no effect on caspase-3 activity (Table 3).

**Figure 4:**
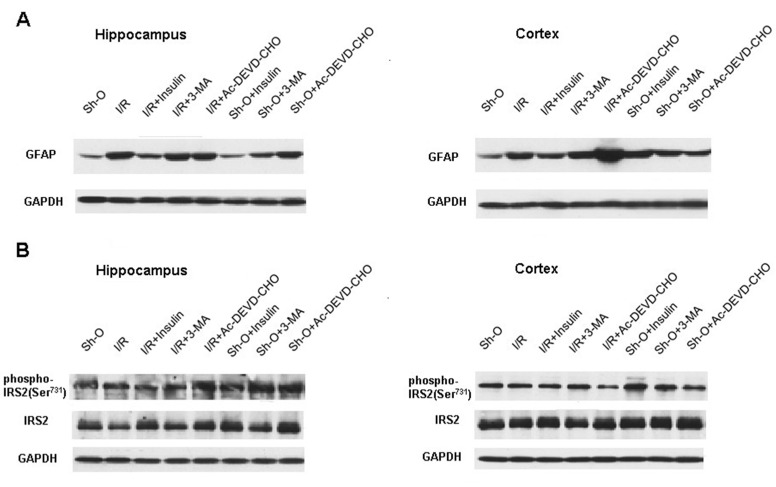
The effect of forebrain ischemia and subsequent reperfusion, insulin (administered intranasally), 3-MA and Ac-DEVD-CHO (administered ICV) on the level of GFAP and pIRS-2 (Ser^731^) in rat hippocampus and frontal cerebral cortex (immunoblots). (**A**) The changes in GFAP level. (**B**) The changes in the level of pIRS-2 (Ser^731^). The quantitative data are summarized in Table 5. Abbreviations used are given in Figure 1.

In order to assess the activation of astroglia during forebrain ischemia and reperfusion in the hippocampus and frontal cerebral cortex, the content of glial fibrillary acidic protein (GFAP) was estimated using the immunoblotting method. Ischemia and reperfusion significantly increased GFAP levels in the hippocampus and frontal cortex (Figure 4, Table 4). However, neither the intranasal administration of insulin nor the ICV administration of autophagy or apoptosis inhibitors to rats exposed to forebrain ischemia and reperfusion significantly diminished the level of GFAP in the hippocampus. At the same time, the GFAP level in the ischemic and reperfused hippocampus ceased to differ significantly from its control level (that is, the level in the hippocampus of sham-operated rats) if insulin was administered to rats (Figure 4, Table 4). It is not possible to exclude that the intranasally administered insulin may significantly decrease the GFAP level in the hippocampus of rats with forebrain ischemia and reperfusion in somewhat different conditions of experiments, such as, for example, if another period of reperfusion is used.

In the frontal brain cortex, high values of GFAP levels, much higher than control values in sham-operated animals, were characteristic of ischemic animals that were not administered any protectors and persisted in ischemic rats treated with intranasal insulin or inhibitors of autophagy or apoptosis.

In the forebrain regions, we studied the effect of ischemia and reperfusion, as well as the administration of insulin and autophagy and apoptosis inhibitors on the intensity of insulin receptor substrate-2 (IRS-2) phosphorylation at Ser^731^. Insulin signaling depends on the phosphorylation of IRS at tyrosine and serine residues. In the brain tissue, phosphorylation of IRS-1 and IRS-2 at tyrosine residues usually increases insulin signaling. But serine phosphorylation of IRS-1 and IRS-2, on the contrary, weakens the insulin effects, and it appears to play an important role in the development of resistance to insulin. It is able to diminish significantly the tyrosine phosphorylation of IRS-1 and IRS-2 [32]. As shown in Figure 4 and Table 4, our experiments did not reveal significant changes in IRS-2 phosphorylation at Ser^731^ in the conditions of our experiments. Neither ischemia and reperfusion nor administration of insulin or inhibitors of autophagy and apoptosis resulted in significant changes in this kind of serine phosphorylation of IRS-2.

**Figure 5:**
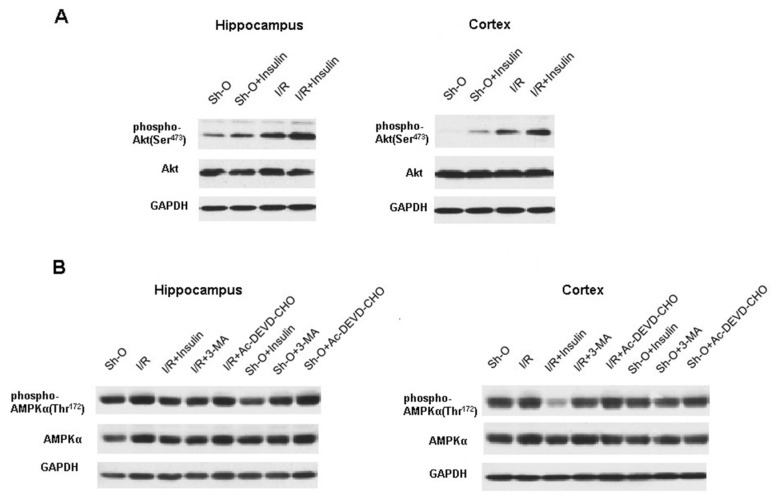
The effect of forebrain ischemia and subsequent reperfusion, insulin (administered intranasally), 3-MA and Ac-DEVD-CHO (administered ICV) on Akt-kinase and AMPK-alpha activities in rat hippocampus and frontal cerebral cortex (immunoblots). (**A**) he activity of Akt-kinase was estimated by the changes in the level of pAkt (Ser^473^); (**B**) the activity of AMPKalpha was estimated by the changes in the level of pAMPK-alpha (Thr^172^) (immunoblots). The quantitative data are summarized in Table 5. Abbreviations used are given in Figure 1.

We were interested in seeing the effect of intranasal administration of insulin and other compounds on the activity of protein kinase B (Akt-kinase) and adenosine monophosphate-activated protein kinase-alpha (AMPK-alpha). As one can see from the data presented in Figure 5 and Table 5, forebrain ischemia and reperfusion activated Akt-kinase in both the hippocampus and the frontal cerebral cortex after the onset of ischemia and subsequent reperfusion for 2 h. Insulin administration before ischemic insult further increased Akt-kinase activity in the hippocampus (*p* < 0.02) and in the frontal cerebral cortex (*p* < 0.05). However, at this early stage of reperfusion, we were unable to reveal the changes in AMPK-alpha activity induced by insulin administration in rats.

At the same time, it was shown that insulin significantly suppressed the AMPK-alpha activity in the hippocampus and frontal cerebral cortex of ischemic rats three days after the beginning of reperfusion (Figure 5, Table 5). In these experiments, the administration of insulin took place before the onset of brain ischemia and daily during reperfusion. In the hippocampus and the frontal cortex, the decrease in the activity of this protein kinase by intranasally administered insulin was quite pronounced and significant. Insulin also caused a significant decrease in AMPK-alpha activity in the hippocampus of sham-operated animals (Figure 5, Table 5).

## 4. Discussion

It is currently believed that it is not possible to achieve success in the treatment of strokes and other ischemic injuries of the brain without understanding the contribution of autophagy to the death of neurons under various damaging effects on the brain [18,19]. Autophagy means “self-eating”, and it includes various catabolic processes, which result in the degradation of long-lived proteins, damaged cells and their organelles, misfolded proteins and other molecules. The products of their degradation are reused to maintain cellular homeostasis. At first, the protective role of autophagy in various organs, including the central nervous system, was studied. It was shown that autophagy activation may protect neurons and other brain cells from the harmful effects of stressors, including brain ischemia and reperfusion. But, at present, ideas about the exclusively protective effect of autophagy on cells of various organs have been revised.

There is extensive evidence that activation of autophagy can lead to neuronal death during ischemia and reperfusion and other brain lesions. That is why it is important to find out if promising neuroprotectors are able to prevent or diminish the autophagic death of brain nerve cells at various injuries. In our studies, it was for the first time shown that administration of insulin to rats with brain ischemic injury prevented or diminished the death of brain nerve cells from activation of autophagy during ischemia and reperfusion (Table 1, Figure 1 and Figure 2). Using ICV administration of 3-MA and Ac-DEVD-CHO, it was found that the death of nerve cells in ischemic and reperfused forebrain regions is due mainly to the activation of autophagy and apoptosis. Insulin administered intranasally normalized the number of live neurons in the CA1 region of the hippocampus, increasing it to the level characteristic for the same brain region of sham-operated rats and thus practically preventing both the autophagic and apoptotic neuronal death in the hippocampal CA1 region. In the ischemic and reperfused frontal brain cortex of rats, the death of neurons was decreased by intranasally administered insulin, but the effect was not as pronounced as in the CA1 region of the hippocampus.

In order to maintain brain homeostasis, the blood–brain barrier restricts the access of large molecules into the brain from systemic circulation. That is why the most effective ways of insulin (and many other proteins) delivery to the brain may be its intranasal or intracerebroventricular (ICV) administrations, as they permit it to bypass the blood–brain barrier. In the case of intranasal administration, insulin may be delivered directly to the brain by a paracellular route along the olfactory or trigeminal nerves [7,8], and the transcellular route of insulin delivery to the brain also exists [8]. At the same time, in the case of ICV administration, insulin is delivered to the brain from cerebrospinal fluid. ICV administration is traumatic as it needs a craniotomy, but it is used in animal experiments. In contrast, intranasal administration of insulin is not traumatic and may be recommended to humans if the protection of brain neurons from neurodegenerative, traumatic or ischemic injury is necessary. Systemic administration is also used to deliver insulin to the brain, but, in this case, much higher doses of insulin are needed to make its injection effective—like 7–8 IU of insulin per rat (20 IU/kg) [33]. It should be also mentioned that in contrast to systemic injection of insulin, its intranasal or ICV administration does not lead to a marked decrease in glucose levels in the blood. The delivery of insulin to the brain takes a short time if intranasal administration is performed. Thus, in one such study, the maximal increase in insulin in rat brains was shown to occur 15 min after its input to nostrils [7]. That is why we used intranasal administration of insulin to rats in our study.

It was shown that the level of one of the main markers of autophagy, LC3B-II, significantly increased in the hippocampus and frontal cortex under the influence of ischemia and reperfusion, while administration of insulin to rats significantly diminished its level in these brain structures (Figure 3, Table 2). The cytosolic form of LC3B (LC3B-I) upon activation of autophagy is converted to the lipidated form—LC3B-II—which plays an important role in the formation of the autophagosomal membranes [34,35]. Previously, in brain cortical neurons, we provided evidence on the ability of insulin to suppress autophagic processes in brain cortical neurons [36]. It was found that oxidative stress activated autophagic processes in these cells, studying its effect on the content of LC3B-II and SQSTM1/p62 in cultivated neurons. When cortical neurons in culture were exposed to hydrogen peroxide, the level of LC3B-II increased significantly, while the level of SQSTM1/p62, on the contrary, decreased [36]. Both effects provide evidence of the activation of autophagic processes in neurons under oxidative stress conditions, as SQSTM1/p62 is degraded in lysosomes upon activation of autophagy, while LC3B-II is, on the contrary, increased when autophagy is activated. In the case of preincubation of cells with insulin, the level of LC3B-II significantly diminished, while the level of SQSTM1/p62 was elevated. These data show that insulin is able to suppress the activation of autophagy in neurons exposed to hydrogen peroxide [36]. This is of interest because oxidative stress is one of the most important causes of neuronal death during ischemia and subsequent reperfusion of the brain. All these data obtained show the ability of insulin to suppress autophagic processes in neurons activated during cerebral ischemia and reperfusion. In this regard, insulin now may be added to the list of substances that have neuroprotective effects, diminishing the intensity of autophagy and the autophagic neuronal death in the ischemic and reperfused brain [18,19,20,21,22,23,24].

Thus, our studies showed for the first time that during forebrain ischemia and reperfusion, intranasally administered insulin is able to inhibit autophagic processes in neurons and to prevent or diminish autophagic neuronal death in two forebrain structures (hippocampal CA1 region and frontal cerebral cortex). Previously, insulin was shown to inhibit autophagic processes in the extraneural organs and cells [37,38]. Last year, a paper was published showing that intranasal administration of insulin to rats with brain trauma inhibits autophagic processes and thus increases the viability of neurons in the brain [39]. This study and our present work provide the first examples of insulin’s ability to inhibit the autophagic processes in neurons and decrease their autophagic death.

According to our data, neurons in the CA1 region of the hippocampus are more sensitive to the effects of ischemia and reperfusion than neurons in the frontal cortex. These results are consistent with the data of other authors (see, for example, [40,41]).

In the present study, it was also shown that in the ischemic and reperfused rat brain, the activity of caspase-3 significantly increased, while intranasally administered insulin inhibited caspase activity in the hippocampus and frontal brain cortex, decreasing it to the level in the same brain regions of sham-operated rats (Table 3). These data show that insulin diminishes the apoptotic death of forebrain neurons. They are consistent with the data obtained by other authors [42,43]. Thus, Russo and co-authors [43] provided evidence that ICV administration of insulin decreased the death from activation of apoptosis and enhanced the number of live neurons in various brain regions, in particular, and pronounced changes were found in the hippocampal CA3 region.

The neuroprotection by intranasally administered insulin is investigated in numerous studies by various authors in Alzheimer’s disease and other neurodegenerative pathologies, but in brain ischemia and reperfusion, predominantly, the protective effect of intranasally administered IGF-1 (but not of insulin) is studied [44,45,46,47]. At the same time, the effect of the administration of exogenous insulin or IGF-1 on autophagic neuronal death in ischemic and reperfused brains has not been investigated yet.

At present, much attention is paid to studies of the activation of the glial cells during brain ischemia and reperfusion [48,49]. We tried to find out whether insulin and inhibitors of autophagy and apoptosis are able to influence the processes of astroglial cell activation. In the hippocampus and frontal cortex, an elevation of GFAP level was shown after global brain ischemia and subsequent three-day reperfusion. An increase in GFAP levels provides evidence of astroglia activation, as it is also one of the manifestations of inflammatory processes [44]. It is to be noted that the elevation of GFAP level may be considered an indicator of gliosis and the formation of glial scars in the brain tissue if the damaging effect of ischemia and reperfusion is strong [48]. But, at the same time, the activation of brain astroglia, if it results in the activation of its phagocytic functions, can lead to an augmentation of the number of living neurons. It was shown that an elevation of the level of GFAP may occur under the influence of a neuroprotector and may be combined with an increase in neuron viability and improvement in the functional state of the brain [49]. Of course, this is only possible if astroglia activation does not lead to irreversible gliosis.

We could not detect significant changes in the level of GFAP in the hippocampus or frontal brain cortex either under the influence of insulin administration or under the influence of autophagy and apoptosis inhibitor administration in rats with brain ischemia and reperfusion (Figure 4, Table 4). At the same time, when insulin was administered, the level of GFAP in the hippocampus decreased so much that it ceased to differ significantly from its level in this brain region of sham-operated rats. Probably, if the conditions of experiments, for example, the time of reperfusion, are changed to a certain extent, it would be possible to detect the significant decrease in GFAP level in the hippocampus of rats with ischemic and reperfused brains by insulin. In the frontal cerebral cortex, a marked increase in the level of GFAP compared to its level in the same brain structure of sham-operated rats was revealed in the course of ischemia and reperfusion in the case of administration of insulin or autophagy and apoptosis inhibitors, as well as in the case of their absence (Figure 4, Table 4).

Insulin signaling depends to a large extent on its regulation by insulin receptor substrates (IRS). There are 6 IRS forms in total. IRS-1 and IRS-2 predominate in various brain regions. Phosphorylation of these substances at tyrosine residues results in the enhancement of insulin action [50,51]. At the same time, these substances are able to inhibit the effects of insulin in brain tissue. These effects are carried out mainly by IRS-1 and IRS-2 phosphorylation at serine residues, which contributes to the appearance of resistance to insulin [51,52]. Phosphorylation of IRS-2 at Ser^731^ has been shown to be significantly increased in nervous tissue in diabetes, contributing to the development of resistance to insulin [51]. But, in our experiments, there was no significant change in IRS-2 phosphorylation at Ser^731^ in the hippocampus or frontal cortex under the influence of intranasally administered insulin or inhibitors of autophagy and apoptosis in rats exposed to brain ischemia and reperfusion (Figure 4, Table 4). If it was revealed that insulin caused a significant increase in the phosphorylation of IRS-2 at Ser^731^ during ischemia and reperfusion in our work, it may be suggested to make a contribution to the appearance of resistance to insulin.

It is interesting to elucidate the mechanism by which insulin, when administered intranasally to rats with forebrain ischemia and reperfusion, diminishes or prevents autophagic and apoptotic death of brain neurons. Among the signaling systems that regulate autophagy processes, an important role is played by AMPK, which is capable of initiating autophagy processes if the accumulation of adenosine monophosphate (AMP) and decrease in ATP level take place, which can occur, in particular, during food insufficiency [53]. AMPK is also activated by other stress factors, such as generated free radicals, increased concentration of calcium ions in the cytosol and others. The protein kinase AMPK, when activated, phosphorylates the protein kinase ULK-1 at Ser^317^ and Ser^777^ [54]. This protein kinase is a part of the complex that initiates autophagic processes. Such phosphorylation of ULK-1 activates this protein kinase and the complex, which results in the activation of autophagy.

This work shows that intranasal administration of insulin to rats exposed to cerebral ischemia and reperfusion inhibits AMPK-alpha activity in the hippocampus and frontal cerebral cortex of rats (Figure 5, Table 5). This protein kinase can initiate autophagy activation in various cells, including neurons. Under normal conditions for the existence of nerve cells and a sufficient amount of food, the level of AMP and, accordingly, the activity of AMPK is relatively low, and the mTORC1 complex is hyperactivated [55,56,57]. On the contrary, with limited food resources or starvation, as well as with various stress and adverse effects on the brain, including brain ischemia and reperfusion, AMPK is activated and autophagic processes are launched [55,56]. The inhibition of AMPK-alpha activity by insulin administered intranasally to rats with forebrain ischemia and reperfusion, demonstrated in this study, undoubtedly makes a major contribution to the ability of insulin to inhibit autophagic processes and diminish the death of forebrain nerve cells caused by the activation of autophagy.

This work also provides evidence that intranasal administration of insulin to rats exposed to brain ischemia and reperfusion causes significant activation of protein kinase B (Akt-kinase) (Figure 5, Table 5), which is able to contribute to the inhibition of autophagic processes due to activation of the mTOR complex 1 (mTORC1) [57]. This complex possesses protein kinase activity and inhibits autophagic processes. mTORC1 is actively involved in the regulation of the intensity of autophagy in mammals. The inhibition of autophagy occurs, in particular, due to the fact that mTORC1 carries out inhibitory phosphorylation at Ser^757^ of the protein kinase ULK-1 [54,56], which, as indicated, is a part of the complex that initiates autophagy and regulates its intensity. The activity of this complex is inhibited by the phosphorylation of ULK-1 at Ser^757^ [54,56].

Thus, Akt-kinase is another protein kinase that, along with AMPK, plays an important role in the regulation of autophagic processes during brain ischemia and reperfusion. The activation of the Akt by insulin makes a major contribution to the prevention by insulin of not only apoptotic, but also autophagic neuronal death in the ischemic and reperfused brain.

It is interesting to note that under the influence of AMPK, activation of autophagy also occurs as a result of its direct inhibitory effect on the mTORC1 complex. And, along with this effect, AMPK phosphorylates and activates tuberous sclerosis protein complex 1/2 (TSC 1/2), which is able to indirectly inhibit mTORC1 [55].

As far as the antiapoptotic effect of insulin administered to rats with brain ischemia and reperfusion is concerned, it depends first of all on the activation of Akt-kinase. This protein kinase, when activated, phosphorylates at Ser^9^ and inactivates GSK-3beta, while activation of this protein kinase leads to mitochondrial dysfunction [58]. In addition, activation of Akt-kinase leads to an increase in antiapoptotic Bcl-2 synthesis and normalization of the Bcl-2/Bax ratio [34,54].

Results showing the ability of intranasal insulin to activate Akt-kinase and to inhibit AMPK-alpha during cerebral ischemia and reperfusion are consistent with the more detailed study on the mechanism of the anti-autophagic and anti-apoptotic effect of insulin in cultured cortical neurons exposed to hydrogen peroxide; these data we obtained and published recently [36]. The ability of insulin to prevent the death of these neurons from the enhancement of autophagy and apoptosis under oxidative stress conditions was shown to depend on the inhibition of AMPK-alpha activity, stimulation of Akt, suppression of GSK-3beta as a result of its phosphorylation at Ser^9^ and on the decrease in Bax/Bcl-2 ratio [36]. It should be mentioned that oxidative stress is one of the main causes of the death of neurons during brain ischemia and reperfusion.

## 5. Conclusions

Using ICV administration of autophagy and apoptosis inhibitors, it was shown that activation of autophagy and apoptosis was the main cause of neuronal death in the CA1 region of the hippocampus and frontal brain cortex of rats with ischemic and reperfused forebrain under the conditions of experiments made. Intranasal administration of insulin (before the onset of ischemia and then daily during 7 days of reperfusion) prevented the death of neurons in the CA1 region of the hippocampus in the ischemic and reperfused brain, making the number of live neurons similar to that in the same brain regions of sham-operated rats. These data provide evidence that intranasal insulin administration to rats with brain ischemia and reperfusion is able to prevent both the apoptotic and the autophagic death of neurons in this brain region. In the frontal brain cortex, insulin administration also diminished the death of neurons, but its effect was not as pronounced as in the hippocampal CA1 region. Along with this neuroprotective effect, intranasal administration of insulin diminished to a large extent the level of LC3B-II in the hippocampus and frontal cerebral cortex, which markedly increased in these brain structures in the course of global forebrain ischemia and reperfusion. The ability of insulin to diminish significantly the caspase-3 activity in the hippocampus and frontal cerebral cortex increased in these forebrain regions after ischemia and reperfusion, was also shown. These data provide evidence that the ability of insulin to prevent both autophagic and apoptotic neuronal death contributes to its neuroprotective effect against brain injury caused by ischemia and reperfusion. It was found that in rats with ischemic and reperfused forebrains, insulin administered intranasally inhibited the activity of AMPK-alpha (protein kinase that initiates and activates autophagy) and activated Akt (protein kinase that inhibits autophagic and apoptotic processes) in the hippocampus and frontal cerebral cortex. The neuroprotective effect of intranasal administration of insulin to rats with forebrain ischemia and reperfusion appears to depend not less on its ability to diminish the autophagic neuronal death than on its ability to decrease the apoptotic death of neurons.

## Figures and Tables

**Table 1:** The effect of administration of insulin and the inhibitors of autophagy (3-MA) and apoptosis (Ac-DEVD-CHO) on the viability of neurons in the hippocampal CA1 region and frontal brain cortex of rats exposed to global ischemia and reperfusion.

Sample	CA1 Region of Hippocampus, the Number of Live Neurons/300 μm	Frontal Cortex, the Number of Live Neurons/Standard Area (300 μm × 300 μm)
Sham-operated rats	46.80 ± 1.42	67.29 ± 1.81
Rats with ischemia and reperfusion (I/R)	27.01 ± 3.23 ^a^	48.76 ± 2.33 ^a^
I/R + 0.5 IU of insulin per rat	46.33 ± 2.9 ^d^	59.38 ± 1.16 ^bd^
I/R + 20 µg 3-MA per rat	38.81 ± 1.53 ^be^	58.01± 2.0 ^be^
I/R + 10 µg Ac-DEVD-CHO per rat	35.33 ± 1.47 ^af^	55.94 ± 1.93 ^bf^
Sham-operated rats + 0.5 IU of insulin per rat	44.65 ± 1.7	65.09 ± 1.77
Sham-operated rats + 20 µg 3-MA per rat	47.60 ± 1.43	65.62 ± 2.06
Sham-operated rat + 10 µg Ac-DEVD-CHO per rat	47.96 ± 0.93	62.71 ± 1.45

Notes: The results are given as the mean ± SEM from data obtained by measuring the number of live neurons in hippocampal CA1 region and frontal brain cortex in 5 separate experiments (in each group of each experiment, the total number of determinations was not less than 57; one rat was used in each experiment). The administration of insulin in a dose of 0.5 IU per rat was performed intranasally 1 h before the onset of ischemia and then daily during 7 days of reperfusion. The administration of inhibitors of autophagy and apoptosis (3-MA and Ac-DEVD-CHO, respectively) was performed 30 min before the onset of ischemia. The differences are significant according to Student’s unpaired *t* test, which compared a and b -- to the number of live neurons in the same brain region of sham-operated rats, a to *p* < 0.001, b to *p* < 0.01, d, e and f to the number of live neurons in the same brain region of rats exposed to ischemia and reperfusion, d to *p* < 0.01, e to *p* < 0.02, f to *p* < 0.05.

**Table 2:** The ratios of LC3B-II/LC3B-I and LC3B-II/GAPDH in the rat hippocampus and frontal cerebral cortex of sham-operated rats and after forebrain ischemia and subsequent reperfusion and the treatment with insulin (administered intranasally), 3-MA and Ac-DEVD-CHO (administered ICV).

Sample	Hippocampus	Frontal Cerebral Cortex
	LC3B-II/LC3B-I (Arbitrary Units)	
Sh-O	0.569 ± 0.065	0.629 ± 0.028
I/R	0.821 ± 0.051 ^a^	0.738 ± 0.02 ^a^
I/R + insulin	0.660 ± 0.047 ^d^	0.626 ± 0.032 ^c^
I/R + 3-MA	0.678 ± 0.034 ^d^	0.656 ± 0.019 ^c^
I/R + Ac-DEVD-CHO	0.687 ± 0.041	0.675 ± 0.025
Sh-O + insulin	0.567 ± 0.04	0.610 ± 0.022
Sh-O + 3-MA	0.561 ± 0.038	0.627 ± 0.033
Sh-O + Ac-DEVD-CHO	0.490 ± 0.052	0.663 ± 0.016
	LC3B-II/GAPDH (arbitrary units)	
Sh-O	0.791 ± 0.099	0.894 ± 0.083
I/R	1.197 ± 0.084 ^a^	1.130 ± 0.066 ^b^
I/R + insulin	0.866 ± 0.095 ^d^	0.917 ± 0.055 ^d^
I/R + 3-MA	0.755 ± 0.086 ^c^	0.934 ± 0.040 ^d^
I/R + Ac-DEVD-CHO	0.887 ± 0.132	1.031 ± 0.046
Sh-O + insulin	0.857 ± 0.101	0.944 ± 0.043
Sh-O + 3-MA	0.795 ± 0.102	0.915 ± 0.049
Sh-O + Ac-DEVD-CHO	0.692 ± 0.092	0.915 ± 0.049

Notes: The data are given as the mean ± SEM of 7–8 experiments performed. Rats were subjected to forebrain ischemia and subsequent reperfusion for 3 days. ICV administration of 3-MA and Ac-DEVD-CHO was performed 30 min before the onset of ischemia. Intranasal administration of insulin in a dose of 0.5 IU per rat was made 1 h before the onset of ischemia and daily in the course of reperfusion. The differences are significant according to the Student’s unpaired *t* test compared a and b to the data obtained in the same brain region of sham-operated rats, a to *p* < 0.02, b to *p* < 0.05; c and d to the data obtained in the same brain region of rats exposed to ischemia and reperfusion, c to *p* < 0.02, d to *p* < 0.05. Abbreviations used are as in Figure 1.

**Table 3:** The level of caspase-3 activity (arbitrary units) in the rat hippocampus and frontal cerebral cortex after brain ischemia and subsequent reperfusion.

Sample	Hippocampus	Frontal Cerebral Cortex
Sh-O	22.59 ± 1.98	25.00 ± 1.42
Sh-O + insulin	24.45 ± 1.53	27.16 ± 1.58
I/R	38.01 ± 5.21 ^a^	37.06 ± 3.62 ^a^
I/R + insulin	24.04 ± 1.96 ^b^	28.36 ± 1.46 ^c^

Notes: The data are given as the mean ± SEM of 7 experiments made. Rats were subjected to forebrain ischemia and subsequent reperfusion for 3 days. Intranasal administration of insulin in a dose of 0.5 IU per rat was carried out 1 h before the onset of ischemia and daily in the course of reperfusion. The differences are significant according to Student’s unpaired *t* test compared a to the data in the same brain region of sham-operated rats, a to *p* < 0.02; b and c to the data in the same brain region of rats exposed to ischemia and reperfusion, b to *p* < 0.02, c-to *p* < 0.05. Abbreviations used are given in Figure 1.

**Table 4:** The ratios of GFAP/GAPDH and pIRS-2 (Ser^731^)/IRS-2 in the rat hippocampus and frontal cerebral cortex of sham-operated rats and after forebrain ischemia and subsequent reperfusion and the treatment with insulin (administered intranasally), 3-MA and Ac-DEVD-CHO (administered ICV).

Sample	Hippocampus	Frontal Cerebral Cortex
	GFAP/GAPDH (Arbitrary Units)	
Sh-O	0.420 ± 0.120	0.305 ± 0.052
I/R	0.948 ± 0.114 ^a^	0.630 ± 0.076 ^a^
I/R + insulin	0.683 ± 0.107	0.647 ± 0.066 ^a^
I/R + 3-MA	0.766 ± 0.062	0.509 ± 0.066 ^b^
I/R + Ac-DEVD-CHO	0.778 ± 0.083	0.750 ± 0.104 ^a^
Sh-O + insulin	0.480 ± 0.080	0.552 ± 0.095 ^b^
Sh-O + 3-MA	0.480 ± 0.050	0.428 ± 0.070
Sh-O + Ac-DEVD-CHO	0.800 ± 0.130	0.494 ± 0.051 ^b^
	pIRS-2 (Ser^731^)/IRS-2 (arbitrary units)	
Sh-O	1.080 ± 0.105	0.440 ± 0.070
I/R	1.214 ± 0.137	0.480 ± 0.090
I/R + insulin	0.770 ± 0.180	0.480 ± 0.100
I/R + 3-MA	1.110 ± 0.280	0.520 ± 0.070
I/R + Ac-DEVD-CHO	0.910 ± 0.190	0.720 ± 0.210
Sh-O + insulin	0.790 ± 0.160	0.520 ± 0.120
Sh-O + 3-MA	1.420 ± 0.280	0.408 ± 0.070
Sh-O + Ac-DEVD-CHO	1.180 ± 0.210	0.400 ± 0.070

Notes: The data are given as the mean ± SEM of 7–8 experiments made. Rats were subjected to forebrain ischemia and subsequent reperfusion for 3 days. ICV administration of 3-MA and Ac-DEVD-CHO was performed 30 min before the onset of ischemia. Intranasal administration of insulin in a dose of 0.5 IU per rat was made 1 h before the onset of ischemia and daily in the course of reperfusion. The differences are significant according to Student’s unpaired *t* test, which compared a and b to the data in the same brain region of sham-operated rats, a to *p* < 0.02, b to *p* < 0.05. Abbreviations used are given in Figure 1.

**Table 5:** The ratios of pAkt (Ser^473^)/Akt and pAMPK-alpha (Thr^172^)/AMPK-alpha in the rat hippocampus and frontal cerebral cortex of sham-operated rats and after forebrain ischemia and subsequent reperfusion and the 4 treatment with insulin (administered intranasally), 3-MA and Ac-DEVD-CHO (administered ICV).

Sample	Hippocampus	Frontal Cerebral Cortex
	pAkt (Ser^473^)/Akt (Arbitrary Units)	
Sh-O	0.173 ± 0.038	0.070 ± 0.012
Sh-O + insulin	0.170 ± 0.036	0.101 ± 0.015
I/R	0.485 ± 0.065 ^a^	0.597 ± 0.085 ^a^
I/R + insulin	0.757 ± 0.024 ^ac^	0.874 ± 0.065 ^ad^
	pAMPK-alpha (Thr^172^)/AMPK-alpha (arbitrary units)	
Sh-O	1.221 ± 0.066	1.034 ± 0.052
I/R	1.229 ± 0.048	1.053 ± 0.055
I/R + insulin	1.013 ± 0.049 ^c^	0.826 ± 0.092 ^d^
I/R + 3-MA	1.251 ± 0.08	1.100 ± 0.071
I/R + Ac-DEVD-CHO	1.310 ± 0.085	1.106 ± 0.058
Sh-O + insulin	1.019 ± 0.055 ^b^	0.916 ± 0.054
Sh-O + 3-MA	1.201 ± 0.069	1.062 ± 0.078
Sh-O + Ac-DEVD-CHO	1.345 ± 0.047	1.072 ± 0.055

Notes: The data are given as the mean ± SEM of 7–8 experiments performed. Intranasal administration of insulin in a dose of 0.5 IU per rat was performed 1 h before the onset of ischemia and daily in the course of reperfusion. Rats were exposed to forebrain ischemia and subsequent reperfusion for 2 h in the case of determination of pAkt (Ser^473^)/Akt ratio; rats were subjected to forebrain ischemia and subsequent reperfusion for 3 days in the case of determination of pAMPK-alpha (Thr^172^)/AMPK-alpha ratio. ICV administration of 3-MA and Ac-DEVD-CHO was performed 30 min before the onset of ischemia. The differences are significant according to Student’s unpaired *t* test, which compared a and b to the data obtained in the same brain region of sham-operated rats, a to *p* < 0.02, b to *p* < 0.05; c and d to the data obtained in the same brain region of rats exposed to ischemia and reperfusion, c to *p* < 0.02, d to *p* < 0.05. Abbreviations used are given as in the Figure 1.

## Data Availability

Data are contained within the article.
